# Comparative Evaluation of Brandy de Jerez Aged in American Oak Barrels with Different Times of Use

**DOI:** 10.3390/foods10020288

**Published:** 2021-01-31

**Authors:** Manuel J. Valcárcel-Muñoz, María Guerrero-Chanivet, M. Valme García-Moreno, M. Carmen Rodríguez-Dodero, Dominico A. Guillén-Sánchez

**Affiliations:** 1 Bodegas Fundador S.L.U. Departamento de Investigación y Desarrollo, C/San Ildefonso, n 3, 11403 Jerez de la Frontera, Cádiz, Spain; m.valcarcel@bodegasfundador.com (M.J.V.-M.); maria.guerreroch@uca.es (M.G.-C.); 2 Departamento de Química Analítica, Facultad de Ciencias, Instituto Investigación Vitivinícola y Agroalimentaria (IVAGRO), Campus Universitario de Puerto Real, Universidad de Cádiz, 11510 Puerto Real, Cádiz, Spain; maricarmen.dodero@uca.es (M.C.R.-D.); dominico.guillen@uca.es (D.A.G.-S.)

**Keywords:** Brandy de Jerez, *Sherry Cask*^®^, oak wood, aroma, ageing

## Abstract

Brandy de Jerez is a European Geographical Indication for grape-derived spirits aged in oak casks that have previously contained any kind of Sherry wine and, therefore, are known as *Sherry Casks^®^*. Wood compounds have a substantial influence in the quality of the brandies that are aged in the barrels. In the cellar, the barrels that have been used for many years to keep Sherry wine or other wine spirits are often used for this purpose. When wooden barrels are used for the first time, they release a large amount of compounds into the liquid contained in them. Such amount decreases over time but casks life cycle has remained unexplored until now. The present work has the aim to study the brandies obtained from the same wine spirit after two years ageing in three differently oak casks: namely new, 7 years of use (4 years containing Oloroso wine and 3 years containing wine spirits) and 32 years of use (8 years containing Oloroso wine and 24 years containing wine spirits). According to the results from our experiments, even after 32 years of use, the wood barrels still contribute to modify the organoleptic characteristics of brandy. Moreover, the brandies aged in used barrels were judged more balanced than those aged in new barrels.

## 1. Introduction

Brandy de Jerez is a spirit produced in the Southern Spanish area known as *Marco de Jerez*, which includes the cities of Jerez de la Frontera, El Puerto de Santa María and Sanlúcar de Barrameda. It is elaborated following the specifications provided by the Technical File of the Geographical Indication under such name [1,2]. This document defines as a grape-derived spirit with a minimum alcoholic strength of 36% vol. (normally between 36–45% vol.), obtained from wine spirits and distillates aged in under 1000 L oak barrels which have been previously seasoned with Sherry wine. The production process follows a traditional dynamic system employed in the Sherry area known as *Criaderas and Solera*.

Brandy de Jerez exhibits a number of specific organoleptic characteristics that make it different from other spirits. Such characteristics are conferred to brandy by the casks where it is aged. This is so because the barrels used to produce Brandy de Jerez must have contained for a certain length of time some type of Sherry wine, i.e., Fino, Amontillado, Oloroso, or Pedro Ximénez. This conditioning process is known as seasoning and every barrel that has undergone such seasoning process according to the rules established by the Technical File that regulates its elaboration [3], is then referred to as *Sherry Cask^®^.*

Naturally, the characteristics of the final product will be influenced by a number of characteristics related to the wooden cask that have an effect on the ageing process [4], namely the geographical origin and botanical species [5,6,7,8,9,10], the container volume [11] and its wood toasting grade [4,7,12]), the temperature and humidity in the cellar, the ageing time [5,13,14,15] and, only in the case of Brandy de Jerez, the seasoning of the barrel [16,17,18,19,20]. The organoleptic characteristics of a particular brandy will be different if it has been aged in a cask with one type of Sherry wine or another. For these reasons, the characteristics of each barrel represent a crucial element that will determine the outcome of the brandy ageing process. Traditionally, American oak (*Quercus alba*) is the wood type that is most often used by cooperage companies in the Sherry area for the manufacturing of barrels. 

As previously said, casks are active contributors to the organoleptic properties of brandy. Wood composition, atmospheric conditions as well as the type of distillate and its alcoholic strength have an influence on the physical-chemical phenomena in which certain compounds from the wood and other components in the distillate are involved. Extraction processes are the most common among such phenomena, but oxidation, esterification, hydrolysis, ethanolysis, Maillard reactions, polymerization, and polycondensation reactions also take place during the ageing process [4,21,22]. Also, some water evaporates and trickles out of the barrel through its pores.

Most of the compounds that are transferred from the wood into the liquid are responsible for the organoleptic profile of the resulting brandy. Wood is mostly composed of polysaccharides (cellulose and hemicellulose) and lignin. This represents around 90% of the total wood matter. The remaining 10% is composed of extractive compounds such as phenolic compounds (polyphenols or simple phenols), fatty acids, alcohols and inorganic substances [22]. The thermal degradation of lignin during the manufacturing of the barrels or its degradation by ethanolysis and hydrolysis during spirit and wine ageing, together with is acid character [23], make it release certain compounds such as vanillin, coniferylaldehyde, syringaldehyde, sinapaldehyde, and cinnamic and benzoic acids into the distillate [4,22]. The degradation of hemicellulose gives place to compounds such as furfural and its derivatives [24,25]. Hydrolysable tannins, such as gallotannins and ellagitannins, are highly soluble in ethanol-water solutions and their transformation into gallic acid or ellagic acid by hydrolysis is very common [22]. Brandy de Jerez also contains other compounds that come from Sherry wine, such as tartaric, lactic, or succinic acid [20]. In those cases, the barrels act as transfer vectors between the Sherry wine that had been previously contained in the cask and the newly ageing distillate [26].

American oak (*Quercus alba*) toasted wood contains between 460 and 3620 µg/g wood of low molecular weight phenols [7]. 745.24 ± 51.28 µg/g wood of phenolic acids, such as ellagic acid, gallic acid, vanillic acid, or syringic acid, 1608.18 ± 346.20 µg/g wood of phenolic aldehydes and a certain amount of volatile compounds that range between 1919.13 and 2660.91 µg/g wood [27]. With repeated use, the amount of these components extracted from the wood and transferred into the ageing spirit becomes gradually smaller compared to the *Sherry Cask*^®^’s first use [28]. Nevertheless, since new *Sherry Casks*^®^ contain a huge amount of extractable compounds, a study on how the repeated use of the same barrels over the years may affect their capacity to enrich the distillates and, therefore, to have an impact on the final product’s organoleptic profile should be of the utmost interest.

Wine makers use the same barrels again and again for many years and only when they are seriously deteriorated or damaged they are finally discarded. This study intends to confirm that even after many years of use, wooden casks can be used to produce brandy. For that purpose, the same wine spirit has been aged for two years in different oak casks: new, 7 years of use (4 years containing Oloroso wine and 3 years containing wine spirits), and 32 years of use (8 years containing Oloroso wine and 24 years containing wine spirits).

## 2. Materials and Methods

### 2.1. Samples

The study was carried out in 500 L wooden barrels (Tonelerías Domecq, Jerez de la Frontera, Spain) made out of American oak (AO) (*Quercus alba*) staves of a medium toasting grade. The toasting procedure was carried out according to the traditional practices in the Sherry area [29].

The Oloroso wine employed for the seasoning the barrels is a white wine fortified at 18% vol. and aged following the traditional oxidation process in the Sherry area, *Criaderas and Solera* system. 

The wine spirit (grapes of the Airén variety) that was aged in all the experiments was supplied by Bodegas Fundador, S.L.U. It had been obtained by column distillation at 77 % vol. and it was hydrated with demineralized water at 65% vol. 

The conditions of the three experiments in this study are specified in Table 1: *New barrels*, *4 + 3 Used barrels* with 7 years of use (4 years containing Oloroso wine and 3 years containing wine spirits) and *8 + 24 Used barrels* with 32 years of use (8 years containing Oloroso wine and 24 years containing wine spirits). For the experiments *4 + 3 Used barrels* and *8 + 24 Used barrels* were emptied and refilled with fresh wine spirit. 

**Figure 1:**
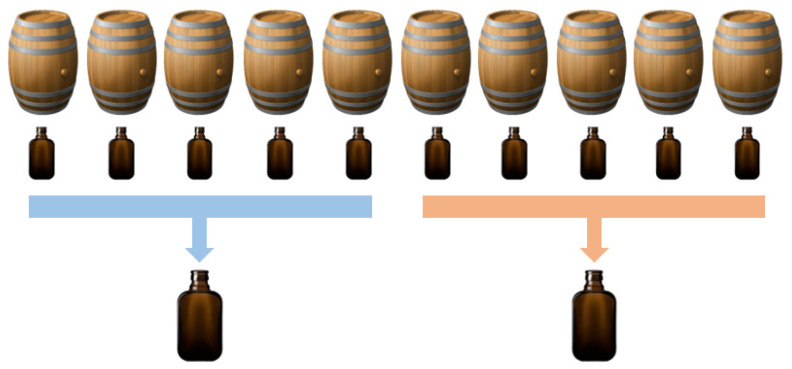
Experimental group of barrels (two set of five barrels) and definition of the sampling plan for the physical-chemical analysis.

Each experimental group comprised ten barrels divided in two set of five barrels each. Individual samples were taken from each barrel and were combined into a pooled sample of each set of five barrels in order to reduce the variability of the barrels (Figure 1). Two pooled samples were obtained (*n* = 2) in each sampling time. This sampling procedure were carried out during two years, at 1, 2, 4, 6, 8, 10, 12, 15, 18, 21, and 24 months, for the three proposed experiences (Table 1). The initial wine spirit was also analyzed. A total of 67 samples were studied. Each sample was analyzed in duplicate.

Since the parameters studied had not been shown a great evolution, only the most relevant results after the first and the second year of ageing have been included in the tables. Ethyl acetate, volatile acids, and Total Polyphenol Index had experimented a significant increase during the ageing, so the complete evolution have been graphically represented.

For the tasting sessions, the two pooled samples of the same experiment were combined into an individual sample of each experiment (n = 1). The initial wine spirit and the samples after 1 year and after 2 years of ageing were tasted. A total of 7 samples were tasted in duplicate in two different sessions.

### 2.2. Reagents

To determine the Total Polyphenol Index (TPI), Folin–Ciocalteu reagent, anhydrous sodium carbonate and gallic acid were purchased from Merck (Darmstadt, Germany).

UHPLC grade acetonitrile from Panreac (Barcelona, Spain), acetic acid from Merck (Darmstadt Germany) were used to prepare the UHPLC phases. The standards for calibration were purchased from Sigma-Aldrich (Saint Louis, MO, USA). Ultrapure water from EMD Millipore (Bedford, MA, USA) was used to prepare the chromatography phases, the reagents and the calibration standards.

### 2.3. Enological Control Parameters 

A pH-Meter Basic 20 (Crison Instruments SA, Barcelona, Spain) was used to measure the pH. The alcoholic strength (% Alcohol by Volume, ABV) was obtained by density measurement of the distillate in a DMA-5000 densimeter (Anton Paar, Ashland, OR, USA). The volatile acids expressed as mg acetic acid/100 mL of 100% vol. alcohol, were measured by means of a segmented flow analyzer AA3 HR Autoanalyzer (Seal Analytical, Norderstedt Stadt, Germany) following the iodide/iodate procedure [30,31,32] according to the method established by the International Organization of Vine and Wine (OIV). The potassium in brandy was determined in mg/L by means of a PinAAcle 900F Atomic Absorption Spectrometer (Perkin Elmer, Boston, MA, USA) and WinLab32 AA (Perkin Elmer, Boston, MA, USA) was the software application used for data acquisition and to perform the data analyses. Each sample was measured in duplicate.

### 2.4. Phenolic Compounds and Furfurals

The phenolic compounds and furfurals were quantified by UHPLC following the method previously established by our research group [33,34]. A Waters Acquity UPLC equipped with a PDA detector and an Acquity UPLC C18 BEH, 100 × 2.1 mm (i.d.) with 1.7 µm particle size (Waters Corporation, Milford, MA, USA) column was employed for the analysis. Nine phenolic compounds (gallic acid, ellagic acid, p-hydroxybenzaldehyde, vanillic acid, vanillin, syringic acid, syringaldehyde, sinapaldehyde, and coniferylaldehyde) and three furanic aldehydes (furfural, 5-methylfurfural, and 5-hydroxymethylfurfural) were identified.

The samples and standards were filtered through 0.22 µm nylon membranes, and they were injected in duplicate. The absorption was determined by UV scanning at between 250 and 400 nm, with a resolution of 1.2 nm. The linear standard curve ranges from 0.1 mg/L to 10 mg/L. The compounds were identified by comparing the retention times and UV-Vis spectra of the sample peaks against those previously obtained from the standards. The results were expressed in mg of compound per 100 mL of 100% vol. alcohol.

### 2.5. Total Polyphenol Index

A Lambda 25 spectrophotometer (Perkin Elmer, Boston, MA, USA) was used to determine the TPI. This instrument was calibrated based on gallic acid aliquots in the range 0–750 mg/L. The total polyphenolic index was measured following the Folin–Ciocalteau method according to the official method established by the International Organization of Vine and Wine (OIV). 0.5 mL of sample, 25 mL of ultrapure water, 2.5 mL of Folin–Ciocalteau reagent and 10 mL of 20% sodium carbonate in strict order were introduced in a 50 mL volumetric flask, and made up to the mark by adding ultrapure water. The dilutions were carried out in duplicate, and the absorbance was measured at 750 nm [35]. Glass cells with a 1 cm optical path were used. The samples were measured in duplicate. The results were expressed in mg gallic acid equivalent (GAE) per 100 mL of 100% vol. alcohol.

### 2.6. Color Measurements

The samples’ color was measured by means of a Lambda 25 spectrophotometer (Perkin Elmer, Boston, MA, USA) at 420 nm, 520 nm and 620 nm absorbance, according to the official method established by the International Organization of Vine and Wine (OIV). Glass cells with a 1 cm optical path were used. Each sample was directly measured in duplicate.

### 2.7. Aldehydes, Acetal, Methanol, Esters, and Higher Alcohols

Aldehydes, acetal, methanol, esters and higher alcohols (also known as fusel alcohols) were quantified by GC-FID. Twenty-one compounds were determined following two different procedures. In both cases, the equipment used was an Agilent 7890B Gas Chromatograph (Agilent Technologies, Santa Clara, CA, USA) coupled with Flame Ionization Detector. 

For the analysis of acetaldehyde, acetaldehyde—diethyl acetal, methanol, ethyl acetate, n-propyl alcohol, 2-butyl alcohol, isobutyl alcohol, n-butyl alcohol, 2-methyl-1-butanol and 3-methyl-1-butanol, the samples were injected in a split mode (split 1:46, 250 °C) into a DB-624 (30 m × 250 µm × 1.4 µm, Agilent Technologies, Santa Clara, CA, USA) column. The oven temperature for the analysis was programmed as follows: 30 °C (30 min), then 6 °C/min to 100 °C (0 min). Temperatures of the injector and the detector were 250 °C and 300 °C, respectively. Nitrogen was used as a carrier at flow of 1.0 mL/min. Data acquisition and analyses were performed using OpenLAB CDS Chemstation (Agilent Technologies, Santa Clara, CA, USA) software.

For the analysis of n-hexanol, 2-phenylethyl alcohol, ethyl lactate, ethyl succinate, ethyl caproate, ethyl caprylate, ethyl caprate, ethyl laureate, ethyl myristate and ethyl palmitate, samples were injected in a splitless mode (1 min, 250 °C) onto CP-WAX 57 CB (25 m × 250 µm × 0.2 µm, Agilent Technologies, Santa Clara, CA, USA) column. The oven temperature program during analysis was as follows: 45 °C (20 min), then 3 °C/min to 170 °C (20 min). Temperatures of the injector and the detector were 250 °C and 300 °C respectively. Nitrogen was used as the carrier gas at a flow of 1.3 mL/min. The data acquisition and analyses were performed using OpenLAB CDS Chemstation (Agilent Technologies, Santa Clara, CA, USA) software.

Standards were made in an ethanol/ultrapure water solution at 40%vol. The linear standard curve of 3-methyl-1-butanol ranges from 1 to 250 mg/100 mL of 100% vol. alcohol. The linear standard curve of methanol, ethyl acetate, n-propyl alcohol, isobutyl alcohol and 2-methyl-1-butanol ranges from 1 to 100 mg/100 mL of 100% vol. alcohol. The linear standard curve of acetaldehyde and acetaldehyde—diethyl acetal ranges from 1 to 50 mg/100 mL of 100% vol. alcohol. The linear standard curve of ethyl lactate ranges from 0.5 to 25 mg/100 mL of 100% vol. alcohol. The linear standard curve of 2-butyl alcohol, n-butyl alcohol, n-hexanol, 2-phenylethyl alcohol, ethyl succinate, ethyl caproate, ethyl caprylate, ethyl caprate, ethyl laureate, ethyl myristate, and ethyl palmitate ranges from 0.1 to 5 mg/100 mL of 100% vol. alcohol. The samples were diluted at 40%vol. with ultrapure water and injected in duplicate. The results were expressed in mg of compound per 100 mL of 100% vol. alcohol.

### 2.8. Tasting Sessions

The tasting sessions took place in a room adequately furnished with individual workspaces to facilitate the concentration and isolation of the tasters [36] at a controlled temperature (20 °C). The 4 tasters were all experts belonging to the staff of Bodegas Fundador, S.L.U. with over 20 years experience in the field and members of the official tasting panel for the Denomination of Origin *Jerez-Xérès-Sherry* and the Geographical Indication *Brandy de Jerez*.

72 h before the tasting sessions the samples were hydrated with demineralized water up to 36% vol. of alcoholic strength, which is the standard alcohol content for the commercial product. 50 mL of each sample was served in black standardized glasses [37], which remained covered by a glass for 10 min in order to stabilize the headspace before the tasting. In each session the set of 7 samples was presented in a random order to the tasters. 

The selected descriptors of odor and flavor (Table 2) were chosen following the indications of the Technical File of the Geographical Indication of *Brandy de Jerez* [1]. Table 2 includes a description of the descriptors and also the odor and flavor patterns employed for the training of the tasters. For the evaluation of the brandies, a numerical scale was used, as defined in ISO 4121:2003 [38]. The pattern (Table 2) would represent a 5 score in the 5-point scale used for the evaluation (5 = very high). Neutral wine alcohol hydrated at 36% vol. would represent a 1 score (1 = absence). The samples were tasted in duplicate in two different sessions.

### 2.9. Statistical Analysis 

Statgraphics 18 software package (Statgraphics Technologies, Inc., The Plains, VA, USA) was employed for ANOVA and Fisher’s Least Significant Difference test. Microsoft Excel 2016 (Microsoft Corp., Redmond, WA, USA) was employed for other statistical parameters.

## 3. Results and Discussion

### 3.1. Enological Control Parameters 

The data corresponding to pH, ABV, volatile acids, and potassium of brandies ageing in new barrels, barrels with 7 years of use and barrels with 32 years of use during the first and second year of ageing are shown in Table 3.

**Figure 2:**
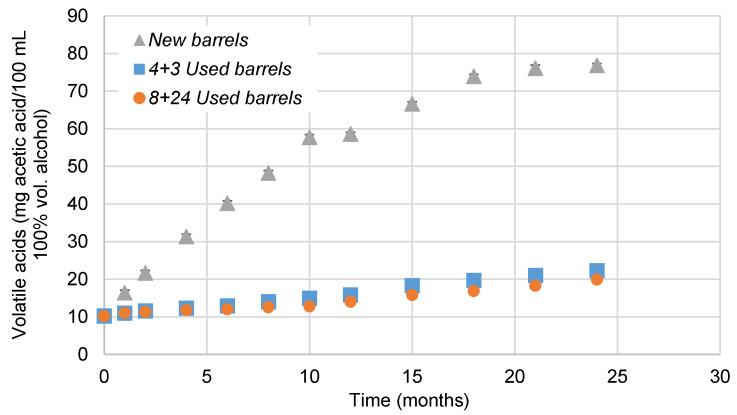
Evolution of volatile acids in brandies aged in *New barrels*, *4 + 3 Used barrels*, and *8 + 24 Used barrels* for two years. When the standard deviation was lower than 0.8 cannot be noticed in the graph.

pH value is around 4, which is characteristics of young brandies [39]. In all the cases studied, their pH decreased over time. In the case of *Used barrels*—*Sherry Casks*^®^—the distillate is enriched with acid compounds from the wood [4,17] or the wine during the ageing process [20]. Some oxidation reactions of the ethanol molecules produce acetic acid during this process too, which also explains why aged brandy contains a greater amount of volatile acids than younger ones (Figure 2). Significant differences associated to used or unused barrels have been observed, but no differences between the brandy aged in *4 + 3 Used barrels* or *8 + 24 Used barrels* were registered. 

The alcoholic strength of all the studied brandies was approximately 65% vol. This percentage decreased when aged in the *Used barrels*, while in the *New barrels* it increased. Since the barrels are not airtight, the evaporation processes that take place inside the barrel are compensated by the perspiration of water molecules to the outside through the wood [40,41]. The volume losses during brandy ageing are shown in Table 4. A greater volume loss was detected in *New barrels* than in *3 + 4* and *8 + 24 Used barrels*. In the *New barrels* experiments, part of the brandy in them was absorbed by the barrel dry wood. This fact explains the higher concentration of compounds such as higher alcohols in brandies kept in *New barrels* compared to that of the brandies in the *Used barrels*.

With regard to potassium, significant differences were found depending on the type of barrel where the brandy was aged. The smallest amount of potassium was found in the brandies aged in *New barrels*. Although new wood would release inorganic compounds into the distillate, such as potassium [42], in this case, seasoning had a greater effect on the final product, since Sherry wines contain inorganic salts such as potassium bitartrate [43]. During the seasoning process, these compounds precipitate and dissolve in the distillate and, as a consequence, the amount of potassium that can be found in brandies aged in *Sherry Casks^®^* is greater than the one detected in *New barrels* without any previous contact with wine.

### 3.2. Phenolic Composition and Total Polyphenol Index of the Aged Brandies

The data corresponding to low molecular weight phenolic compounds content as determined by means of UHPLC in the two-year aged brandies are indicated in Table 5 as mg per 100 mL of 100% vol. alcohol. All the compounds in the study exhibited the same trend: significant differences were observed depending on the barrel type, but no differences were noted between the brandy aged in *4 + 3 Used barrels* and *8 + 24 Used barrels*, excluding the content of syringic acid, vanillin and syringaldehyde. Except for furfural, all of the above mentioned compounds could not be detected in the initial wine spirit, since they are provided by the wood or the wine in the case of seasoned barrels, and they are easily and generally found in brandies aged in wood [4,17,19,20,21,44]. When wood is used for the first time, a large amount of these compounds are transferred into the liquid [45]. Therefore, the brandy held in *New barrels* contains a larger concentration of them than those held in *Used barrels*, because these wood compounds had already been extracted in large quantities during the previous use. From the tasters’ point of view this high concentration of wood compounds might be perceived as a sort of ‘aggressive’ taste, as described in Section 3.5. On the other hand, *Used barrels* release into the distillate Sherry wine compounds that provide the brandy with rather pleasant organoleptic characteristics. Although the phenolic compounds concentration levels detected in the brandies aged in *8 + 24 Used barrels* was slightly lower than in those brandies aged in *4 + 3 Used barrels*, such difference, that could be explained by a lower availability of such compounds in older barrels, could not be regarded as relevant.

With regard to the concentration of the phenolic acids (gallic acid, vanillic acid, syringic acid, and ellagic acid) their values in the different brandies follow the expected pattern (Table 5). Thus, their concentration increased over the ageing period and greater concentrations were observed in the brandies aged in *New barrels* than in those aged in *3 + 4 Used barrels* or *8 + 24 Used barrels*, because of the lower availability of these compounds. Phenolic aldehydes as p-hydroxybenzaldehyde, vanillin, syringaldehyde, coniferylaldehyde, and sinapaldehyde resulting from the thermal degradation of lignin [23,25,46], were found in all the brandies studied (Table 5). Significant amounts of 5-hydroxymethylfurfural, furfural and 5-methylfurfural were also detected with similar content trends in all the experiments (Table 5).

**Figure 3:**
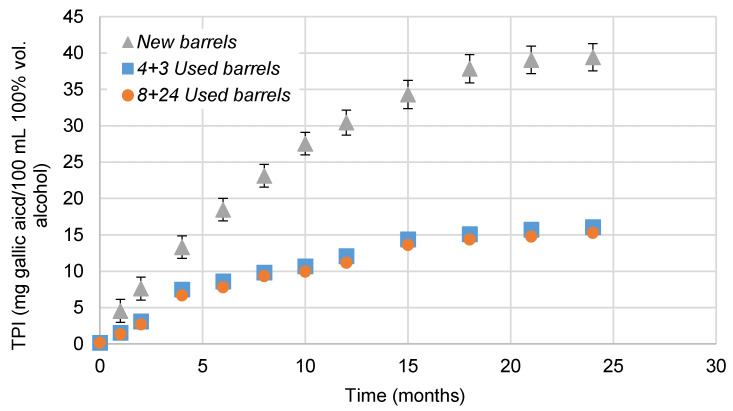
Evolution of Total Polyphenol Index (TPI) in brandies aged in *New barrels*, *4 + 3 Used barrels* and *8 + 24 Used barrels* for two years, expressed in mg gallic acid/100 mL of 100% vol. alcohol. The standard deviation between *4 + 3 Used barrels*-aged brandies and *8 + 24 Used barrels*-aged brandies was lower than 0.7 and cannot be noticed in the graph.

The data corresponding to the TPIs of the studied brandies, expressed in mg GAE/100 mL of 100% vol. alcohol, can be seen in Figure 3. As expected, TPI increased with ageing in all the cases. However, depending on the wood previous usage, it did so in a lesser or greater extent. Thus, the TPI closely followed a similar evolution pattern as the one for low molecular weight phenolic compounds that has been previously discussed [47], since the amount of phenolic compounds available in the wood and, therefore, their concentration in the aged brandy also depend on whether the wood has been previously used or not. 

In this way, when wood is used for the first time (*New barrels*) it releases a much larger quantity of compounds into the distillate than when the wood has been previously seasoned. Nevertheless, a close look at Figure 3 and Table 5 let us see that no significant differences between *4 + 3 Used barrels* and *8 + 24 Used barrels* were noted. All in all, it can be confirmed that, regardless of the length of time that a particular barrel may have been used, typically seasoned barrels will release similar levels of phenolic compounds into the brandy. In fact, even after 32 years of use, barrels will continue to provide brandies with the compounds that confer this spirit with its characteristic and highly appreciated organoleptic profile. 

### 3.3. Chromatic Characteristics

The samples were measured for the following color absorbances: A420, A520 and A620. As expected, the color intensity of the brandies increased with ageing (Table 6). 

The distillates aged in *New barrels* presented the greatest intensity growth. The absorbance at 420 nm, which corresponds to the yellow zone, was higher than the absorbance at 520 nm and 620 nm in all the cases studied. Since the color of the brandy is closely related to the presence of phenolic compounds in the distillate, the increment in A420, A520 and A650 intensity would be related to the ageing process in the wood barrels and to the subsequent extraction and oxidation reactions that take place between the compounds that are being extracted from the wood and those already present in the distillate [44,46,48]. 

Some studies associate the increment in the yellow shade intensities (A420) with the oxidation of the ellagitannins from the wood [49] and with the condensation reactions among tannins in the presence of acetaldehyde and phenolic aldehydes [44]. The intensity of the A420 shade as measured in the experimental brandies in the study presented significant differences that could be associated to the type of barrel used for the ageing of the spirit. Thus, the brandies aged in *New barrels* registered the highest values for this parameter, while those brandies that had been aged in *8 + 24 Used barrels* exhibited the lowest values, i.e., the least intense A420 shade. Hence, it can be concluded that even though this shade intensity increased in all the cases, the brandies that had been aged in *4 + 3 Used barrels* or *8 + 24 Used barrels* presented a paler yellow tone than those brandies aged in *New barrels*.

### 3.4. Aldehydes, Acetal, Methanol, Esters, and Higher Alcohols

Regarding acetaldehyde and acetaldehyde—diethyl acetal, their content did not experiment any marked evolution during the ageing period (Table 7). This is due to the equilibrium that affects these two compounds, influenced by alcoholic strength and pH. Acetaldehyde stabilized its presence during the second year of brandy ageing [50], when its losses due to evaporation and its oxidation into acetic acid are compensated by the concentration of the compound caused by the ethanol evaporation and the perspiration of the water through the wood [40,41] (Table 4). It can be seen in Table 7 that both compounds do not follow a clear evolution.

A similar slight evolution was also observed in the methanol concentration. This is a volatile compound that may evaporate during ageing, but its concentration is also influenced by ethanol evaporation and water perspiration [40,41], thus, its concentration could increase as a consequence of a total volume decrease (Table 4).

**Figure 4:**
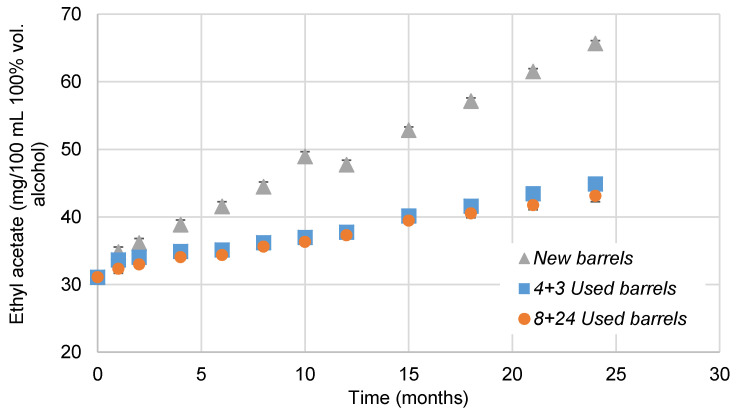
Evolution of ethyl acetate in brandies aged in *New barrels*, *4 + 3 Used barrels* and *8 + 24 Used barrels* for two years, expressed in mg/100 mL of 100% vol. alcohol. When the standard deviation was lower than 0.9 cannot be noticed in the graph.

Ethyl acetate is the compound in these families of volatile substances that most increased its concentration with the ageing process (Figure 4). Its initial content in the wine spirit is determined by the quality of the distilled wine and the type of still or distillation column used. Ethyl acetate is involved in the esterification reactions between the acetic acid (formed during the ageing) and the ethanol. The content levels of this compound in *New barrels* are higher than in *Used barrels*. In recent studies published by our research group [34], it was demonstrated that wood has the capacity to release acetic acid into the distillate, thus increasing its content in the liquid with ageing. After the esterification of the acetic acid has been completed, the amount of ethyl acetate in brandy increases. Significant differences were observed attending to the usage conditions of the barrel, but there were no differences between the brandy aged in *4 + 3 Used barrels* or *8 + 24 Used barrels*.

In relation to the higher alcohols (n-propyl alcohol, 2-butyl alcohol, isobutyl alcohol, n-butyl alcohol, 2-methyl-1-butanol, 3-methyl-1-butanol, n-hexanol and 2-phenylethyl alcohol), slight increments were observed in most of the brandies. Although in some cases, their content level remained invariable. These compounds are not influenced by the wood, since they come from the distillate. They hardly evaporate through the wood pores, because their molecular volume is larger than water or ethanol’s. Thus, their concentration increment could be attributed to the ethanol evaporation and water perspiration during the process [40,41]. After the two years of ageing, a high total amount of higher alcohols was determined in those brandies aged in *New barrels*. This could be explained by the considerable loss of volume that took place over the experiment (Table 4). 

The esters (ethyl lactate, ethyl succinate, ethyl caproate, ethyl caprylate, ethyl caprate, ethyl laureate, ethyl myristate, and ethyl palmitate) either remained stable or increased slightly. In general, their concentration is affected by hydrolysis, but this reaction rarely takes place during the ageing process, since pH and alcoholic strength values remain almost stable. Similarly to the higher alcohols, esters’ concentration increment was a result of the ethanol evaporation and water perspiration during the process (Table 4) [40,41].

The amount of ethyl esters that come from organic acids, such as lactic acid and succinic acid, is larger in *4 + 3* and *8 + 24 Used barrels* than it is in *New barrels* due to the seasoning of the casks, since the presence of these compounds in brandies is explained by the organic acids content in wine (lactic acid and succinic acid), which are involved in the esterification reactions that take place in the liquid over the brandy ageing process. Naturally, their concentration levels were higher in those brandies that had been aged in *3 + 4 Used barrels* than it was in *8 + 24 Used barrels* because of the lower availability of these compounds after the repeated use of each barrel. Thus, significant differences were registered between the three experiments. Furthermore, it should be noted that the concentration of esters derived from fatty acids also increased during the ageing process as a consequence of volume losses. Nevertheless, these compounds were found in very low concentrations in all the Brandies studied here and hardly any differences could be observed between them. 

### 3.5. Tasting Results

According to the tasting sessions carried out, the best perceived descriptors were fruity and vinous notes with medium-high intensity values (between 3 and 5 in a 5-point scale). The ageing notes (vanilla, toasted/caramel, spicy/aniseed, oxidative sweetness, and oak) were evaluated with scores between 1 and 3 points, with the highest values awarded to the brandies that had already been aged for two years in new oak barrels. All of them were characterized by their complex aromatic profile, soft and balanced sensations in the oral cavity, with a remarkable presence of the alcoholic component.

The highest intensity of vinous note was found in the two-year aged brandies, while the lowest intensity was attributed to the young wine spirits. Similar scores were awarded to aromatic complexity, vanilla, toasted/caramel, spicy/aniseed, softness, oxidative sweetness and oak notes. On the other hand, in terms of flavor balance, 2-year brandies were scored higher by the tasters than the other two younger samples. As expected, fruity character and alcoholic note decreased significantly with longer ageing times. The differences between the aromatic notes of wine, vanilla, toasted/caramel and spicy/aniseed, as well as for the woody note and the balance in the flavor evaluation, were confirmed to be associated to wood age and usage. Thus, when the brandy was aged in new oak barrels, the samples were less vinous and balanced and presented higher notes of vanilla, toasted/caramel and oak than the others brandies. When wood is used for the first time, a large amount of phenolic compounds are transferred into the liquid [45], as can be seen in Section 3.2. It should be highlighted that the 4 tasters associated the highest flavor notes of oak, that had been attributed to the brandies aged in *New Barrels*, with astringent sensations and ‘aggressiveness’. The tasters related the ‘aggressive’ term with the gustatory evaluation: high alcoholic character, high bitter oak notes, low smoothness and low oxidative sweetness, which is the opposite of a balanced product. However, they did not establish such association in the case of the brandies aged in used casks. Regarding these descriptors, no big differences were perceived between aged spirits in *4 + 3 Used barrels* and *8 + 24 Used barrels* according to the tasters’ opinion. The spicy/aniseed note is the only one that was perceived at different intensity between the three types of casks. Thus, the tasters associated the highest values to the samples aged in *New barrels* and the lowest for the samples aged in *8 + 24 Used barrels*. The softness sensation perceived in the oral cavity was higher in brandies aged in *4 + 3 Used barrels* than in brandies aged in *8 + 24 Used barrels*, followed by the spirits aged in *New barrels*. 

**Figure 5:**
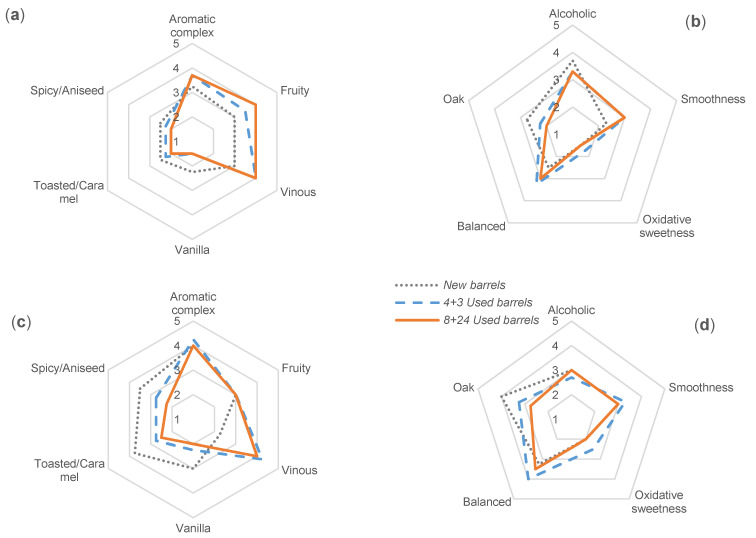
Radar charts of the tasting results of brandies aged for 1 and 2 years in barrels with different previous usage: *New barrels*, *4* + *3 Used barrels*, *8* + *24 Used barrels*. (**a**) Odor evaluation after 1 year of ageing; (**b**) Flavor evaluation after 1 year of ageing; (**c**) Odor evaluation after 2 years of ageing; (**d**) Flavor evaluation after 2 years of ageing (Legend in Figure 5d).

When focusing on mean values (Figure 5), the differences in the taste profile of the brandies aged in *Used barrels* with respect to the Brandies aged in *New barrels* grows wider as wood contact time increases. 

## 4. Conclusions

There is a clear difference between brandies aged in *New barrels* and those aged in used or seasoned barrels. When barrels are used for the first time, they release much larger amounts of wood compounds into the distillate than in later uses of the same barrel. However, once the barrel has been used, no significant differences can be observed in relation to the number of years of use. In fact, although slightly higher levels of volatile acids, phenolic compounds, A420, A520, and A620, as well as aldehydes, acetal, methanol, esters, and higher alcohols have been detected in the brandies aged in *4 + 3 Used barrels* compared to *8 + 24 Used barrels*, such differences were irrelevant in most cases.

The tasters described as ‘aggressive’ the brandies aged in the *New barrels*, that released a high number of compounds. Moreover, the final product was not considered as well balanced as those obtained from *Used barrels*. On the other hand, in most cases the taster did not perceived differences between brandies aged in *4 + 3* or *8 + 24 Used barrels*.

It has been confirmed that even after 32 years of use the barrels’ wood would still contribute to the organoleptic properties of brandy. Obviously, *Used barrels* do not yield the same amount of wood compounds into the distillates, but, attending to the organoleptic properties of the final product, this could be considered a positive factor. According to the main results of this study, it can be concluded that wooden barrels that have been used for a high number of years can still be used for ageing distillates, providing them with different organoleptic characteristics than *New barrels*.

## Figures and Tables

**Table 1:** Experimental conditions under study.

Experience	Previous Use of the Barrels		
	Sherry Seasoning		Brandy Ageing
	Type	No. of Years	No. of Years
*New barrels*	None	No seasoning	No previous use
*4 + 3 Used barrels*	Oloroso	4	3
*8 + 24 Used barrels*	Oloroso	8	24

**Table 2:** Odor and flavor descriptors and patterns used for the training of the tasters.

Descriptor	Definition	Pattern
**Odor**		
Aromatic complexity	Diversity of positive aromatic sensations.	5 years old brandy, produced with pot stills, hydrated at 36% vol.
Fruity	Fruit aromas characteristic of the grape varieties used to produce the wine and its distillate (apple, pear, banana, pineapple, tropical fruits, etc.).	Wine spirit from pot stills hydrated at 36% vol., with a level of fatty acids ethyl esters and higher alcohols acetates of 35 mg/L.
Vinous	‘Memories’ of the original wine used to produce the distillate and compounds acquired during ageing in *Sherry Casks^®^*.	5 years old brandy, produced with pot stills, hydrated at 36% vol.
Vanilla	Compounds released during lignin degradation. ‘Noble’ wood sensations.	Wine spirit hydrated at 36% vol. with lignin vanilla addition.
Toasted/caramel	Sensations of toasted wood.	Wine spirit hydrated at 36% vol. after adding hydroalcoholic medium toasted American oak extract.
Spicy/aniseed	Sensations of non-toasted wood and the characteristic smell of medium chain wine ethyl esters (C6).	Wine spirit hydrated at 36% vol. after adding hydroalcoholic raw American oak extract and wine lees.
**Flavor**		
Alcoholic	Burning sensation in the oral cavity.	Neutral wine alcohol hydrated at 36% vol., with addition of distillation heads and tails, with a level of volatile compounds of 500 mg/100 mL of 100% vol. alcohol.
Smoothness	Pleasant as it goes down the throat.	5 years old brandy hydrated at 36% vol.
Oxidative sweetness	Velvety sensation in the oral cavity.	5 years old brandy hydrated at 36% vol., after adding 3 g/L of rectified concentrate grape must.
Equilibrium	General evaluation of all the sensations, from the first contact with the liquid until it is swallowed: structured, absence of astringency or bitterness, etc.	8 years old brandy Solera Gran Reserva hydrated at 36% vol.
Oak/woody	Wood sensation; seasoned, unseasoned.	5 years old brandy hydrated at 36% vol., after adding hydroalcoholic medium toasted American oak extract.

**Table 3:** pH, alcoholic strength (% Alcohol by Volume, ABV), volatile acids (mg acetic acid/100 mL of 100% vol. alcohol) and potassium (mg/L) of brandies aged for one or two years in *New barrels*, *4 + 3 Used barrels*, and *8 + 24 Used barrels*.

	Initial	*New Barrels*		*4 + 3 Used Barrels*		*8 + 24 Used Barrels*	
		1 Year Ageing	2 Years Ageing	1 Year Ageing	2 Years Ageing	1 Year Ageing	2 Years Ageing
pH	4.52	4.33 ± 0.01 ^a^	4.20 ± 0.03 ^a^	4.12 ± 0.03 ^b^	3.99 ± 0.01 ^b^	4.10 ± 0.02 ^b^	4.00 ± 0.03 ^b^
Volatile acids	10.3	58.6 ± 0.4 ^a^	76.9 ± 0.4 ^a^	15.9 ± 0.5 ^b^	22.3 ± 0.7 ^b^	14.0 ± 0.8 ^b^	20.0 ± 0.4 ^c^
Alcoholic strength	65.27	65.36 ± 0.04 ^a^	65.57 ± 0.02 ^a^	65.13 ± 0.03 ^b^	65.15 ± 0.04 ^b^	65.14 ± 0.03 ^b^	65.16 ± 0.00 ^b^
Potassium	n.d.	5.90 ± 0.30 ^a^	8.00 ± 0.15 ^a^	15.50 ± 0.71 ^b^	17.00 ± 1.41 ^b^	7.00 ± 0.00 ^a^	8.50 ± 0.71 ^a^

Mean values ± standard deviation (*n* = 2); significant differences (*p* < 0.05) of a particular parameter in the same ageing year are indicated by different letters; n.d.: Not detected.

**Table 4:** Brandy volume loss after two years of ageing.

Experiment	*New Barrels*	*4 + 3 Used Barrels*	*8 + 24 Used Barrels*
Average volume loss after the first year (L/500 L barrel)	28.1 ± 3.4 ^a^	14.1 ± 3.3 ^b^	14 ± 3.4 ^b^
Average volume loss after the second year (L/500 L barrel)	13.7 ± 3.3	13 ± 3.2	14.6 ± 3.4
Average percentage of volume loss per year (%)	4.2 ± 0.7 ^a^	2.7 ± 0.7 ^b^	2.9 ± 0.7 ^a,b^

Data are mean values ± standard deviation (*n* = 2); values in the same row with different letters are significantly different (*p* < 0.05).

**Table 5:** Phenolic compounds content (mg/100 mL of 100% vol. alcohol) in brandies aged in *New barrels*, *4 + 3 Used barrels*, and *8 + 24 Used barrels* after the first and the second year of ageing.

	Initial	*New Barrels*		*4 + 3 Used Barrels*		*8 + 24 Used Barrels*	
		1 Year Ageing	2 Years Ageing	1 Year Ageing	2 Years Ageing	1 Year Ageing	2 Years Ageing
Gallic acid	n.d.	0.83 ± 0.05 ^a^	1.02 ± 0.05 ^a^	0.29 ± 0.02 ^b^	0.47 ± 0.03 ^b^	0.22 ± 0.02 ^b^	0.36 ± 0.02 ^b^
Vanillic acid	n.d.	0.52 ± 0.02 ^a^	0.60 ± 0.06 ^a^	0.06 ± 0.00 ^b^	0.09 ± 0.01 ^b^	0.05 ± 0.01 ^b^	0.08 ± 0.01 ^b^
Syringic acid	n.d.	0.47 ± 0.02 ^a^	0.58 ± 0.02 ^a^	0.14 ± 0.01 ^b^	0.24 ± 0.02 ^b^	0.08 ± 0.01 ^c^	0.12 ± 0.01 ^c^
Ellagic acid	n.d.	2.48 ± 0.05 ^a^	2.90 ± 0.05 ^a^	1.52 ± 0.03 ^b^	1.97 ± 0.03 ^b^	1.57 ± 0.07 ^b^	1.90 ± 0.08 ^b^
p-Hydroxybenzaldehyde	n.d.	0.06 ± 0.01	0.05 ± 0.01	n.d.	n.d.	n.d.	n.d.
Vanillin	n.d.	0.64 ± 0.02 ^a^	0.78 ± 0.03 ^a^	0.09 ± 0.01 ^b^	0.15 ± 0.01 ^b^	0.06 ± 0.01 ^b^	0.09 ± 0.01 ^c^
Syringaldehyde	n.d.	1.39 ± 0.07 ^a^	1.59 ± 0.07 ^a^	0.24 ± 0.01 ^b^	0.39 ± 0.02 ^b^	0.12 ± 0.01 ^b^	0.21 ± 0.01 ^c^
Coniferylaldehyde	n.d.	1.60 ± 0.05 ^a^	1.57 ± 0.04 ^a^	0.05 ± 0.00 ^b^	0.07 ± 0.01 ^b^	0.03 ± 0.00 ^b^	0.05 ± 0.01 ^b^
Sinapaldehyde	n.d.	2.59 ± 0.09 ^a^	2.99 ± 0.07 ^a^	0.03 ± 0.00 ^b^	0.06 ± 0.01 ^b^	0.03 ± 0.00 ^b^	0.05 ± 0.01 ^b^
5-Hydroxymethylfurfural	n.d.	1.63 ± 0.06 ^a^	1.60 ± 0.06 ^a^	0.04 ± 0.01 ^b^	0.06 ± 0.01 ^b^	0.02 ± 0.00 ^b^	0.03 ± 0.01 ^b^
Furfural	0.083	3.72 ± 0.17 ^a^	4.04 ± 0.12 ^a^	0.18 ± 0.01 ^b^	0.23 ± 0.01 ^b^	0.16 ± 0.01 ^b^	0.20 ± 0.01 ^b^
5-Methylfurfural	n.d.	0.54 ± 0.02	0.52 ± 0.03	n.d.	n.d.	n.d.	n.d.

Data are mean value ± standard deviation (*n* = 2); significant differences (*p* < 0.05) of a particular parameter in the same ageing year are indicated by different letters; n.d.: Not detected.

**Table 6:** Chromatic characteristics of the brandies studied after 1 and 2 years of ageing.

	Initial	*New Barrels*		*4 + 3 Used Barrels*		*8 + 24 Used Barrels*	
		1 Year Ageing	2 Years Ageing	1 Year Ageing	2 Years Ageing	1 Year Ageing	2 Years Ageing
A420	n.d.	0.327 ± 0.020 ^a^	0.410 ± 0.018 ^a^	0.189 ± 0.008 ^b^	0.268 ± 0.008 ^b^	0.122 ± 0.007 ^c^	0.198 ± 0.006 ^c^
A520	n.d.	0.053 ± 0.004 ^a^	0.086 ± 0.004 ^a^	0.041 ± 0.001 ^b^	0.065 ± 0.001 ^b^	0.028 ± 0.003 ^c^	0.042 ± 0.003 ^c^
A620	n.d.	0.012 ± 0.001 ^a^	0.015 ± 0.001 ^a^	0.011 ± 0.001 ^a^	0.016 ± 0.000 ^b^	0.008 ± 0.000 ^b^	0.011 ± 0.000 ^c^

Data are mean value ± standard deviation (*n* = 2); significant differences (*p* < 0.05) of a particular parameter in the same ageing year are indicated by different letters; n.d.: Not detected.

**Table 7:** Aldehydes, acetal, methanol, esters and higher alcohols contents (mg/100 mL of 100% vol. alcohol) in the brandies studied.

	Initial	*New Barrels*		*4 + 3 Used Barrels*		*8 + 24 Used Barrels*	
		1 Year Ageing	2 Years Ageing	1 Year Ageing	2 Years Ageing	1 Year Ageing	2 Years Ageing
Acetaldehyde	21.8	23.8 ± 0.5 ^a^	24.1 ± 0.6 ^a^	21.9 ± 0.6 ^b^	22.0 ± 0.3 ^b^	21.9 ± 0.3 ^b^	21.9 ± 0.3 ^b^
Diethyl acetal	28.8	31.3 ± 0.3 ^a^	32.1 ± 0.3 ^a^	29.4 ± 0.3 ^b^	29.5 ± 0.7 ^b^	29.3 ± 0.4 ^b^	29.4 ± 0.5 ^b^
Methanol	68.0	66.2 ± 0.3 ^a^	66.9 ± 0.3	67.4 ± 0.4 ^a,b^	68.1 ± 0.2	67.9 ± 0.7 ^b^	68.0 ± 0.7
Ethyl acetate	31.1	47.7 ± 0.7 ^a^	65.7 ± 0.4 ^a^	37.8 ± 0.5 ^b^	44.9 ± 0.6 ^b^	37.3 ± 0.5 ^b^	43.1 ± 0.9 ^b^
**Higher alcohols**							
N-propyl alcohol	33.2	37.3 ± 0.2 ^a^	37.8 ± 0.1 ^a^	34.2 ± 0.6 ^b^	34.2 ± 0.6 ^b^	34.0 ± 0.5 ^b^	34.1 ± 0.4 ^b^
2-butyl alcohol	0.5	0.5 ± 0.1	0.5 ± 0.0	0.5 ± 0.0	0.5 ± 0.0	0.5 ± 0.0	0.5 ± 0.0
Isobutyl alcohol	33.2	33.3 ± 0.1	37.2 ± 0.2 ^a^	34.0 ± 0.6	34.5 ± 0.1 ^b^	34.4 ± 0.7	34.4 ± 0.9 ^b^
n-butyl alcohol	2.1	2.3 ± 0.1 ^a^	2.6 ± 0.0 ^a^	2.2 ± 0.0 ^b^	2.2 ± 0.0 ^b^	2.2 ± 0.0 ^b^	2.2 ± 0.0 ^b^
2-methyl-1-butanol	40.3	46.5 ± 0.5 ^a^	50.5 ± 0.2 ^a^	41.4 ± 0.6 ^b^	41.8 ± 0.1 ^b^	40.9 ± 1.0 ^b^	41.4 ± 0.4 ^b^
3-methyl-1-butanol	182.6	184.5 ± 0.0	185.9 ± 0.0	186.3 ± 0.8	188.1 ± 1.1	186.7 ± 1.8	188.3 ± 1.5
N-hexanol	2.8	2.8 ± 0.0	2.9 ± 0.0	2.8 ± 0.0	2.8 ± 0.0	2.8 ± 0.0	2.8 ± 0.0
2-phenylethyl alcohol	0.6	0.6 ± 0.0	0.7 ± 0.0 ^a^	0.6 ± 0.0	0.6 ± 0.0 ^b^	0.6 ± 0.0	0.6 ± 0.0 ^b^
*Total*	295.3	308.1 ± 0.6	318.0 ± 0.1 ^a^	302.0 ± 2.5	304.7 ± 1.8 ^b^	302.2 ± 3.0	304.4 ± 2.3 ^b^
**Ethyl esters from organic acids**							
Ethyl lactate	13.2	13.4 ± 0.0 ^a^	13.5 ± 0.0 ^a^	14.1 ± 0.1 ^b^	14.3 ± 0.1 ^b^	13.9 ± 0.1 ^b^	14.0 ± 0.1 ^c^
Ethyl succinate	1.1	1.1 ± 0.0 ^a^	1.1 ± 0.0 ^a^	1.8 ± 0.1 ^b^	2.0 ± 0.1 ^b^	1.5 ± 0.1 ^c^	1.5 ± 0.1 ^c^
*Total*	14.3	14.5 ± 0.1 ^a^	14.6 ± 0.0 ^a^	15.9 ± 0.3 ^b^	16.5 ± 0.1 ^b^	15.3 ± 0.1 ^c^	15.6 ± 0.1 ^c^
**Ethyl esters from fatty acids**							
Ethyl caproate	0.2	0.3 ± 0.0	0.3 ± 0.0	0.3 ± 0.0	0.3 ± 0.0	0.3 ± 0.0	0.3 ± 0.0
Ethyl caprylate	1.4	1.4 ± 0.0 ^a^	1.5 ± 0.0	1.5 ± 0.0 ^b^	1.5 ± 0.0	1.5 ± 0.0 ^b^	1.5 ± 0.0
Ethyl caprate	1.0	1.0 ± 0.0	1.0 ± 0.0 ^a^	1.0 ± 0.0	1.1 ± 0.0 ^b^	1.0 ± 0.0	1.1 ± 0.0 ^b^
Ethyl laureate	0.2	0.2 ± 0.0	0.2 ± 0.0	0.2 ± 0.0	0.2 ± 0.0	0.2 ± 0.0	0.3 ± 0.0
Ethyl myristate	0.0	0.1 ± 0.0	0.1 ± 0.0	0.1 ± 0.0	0.1 ± 0.0	0.1 ± 0.0	0.1 ± 0.0
Ethyl palmitate	0.4	0.4 ± 0.0	0.4 ± 0.0	0.4 ± 0.0	0.4 ± 0.0	0.4 ± 0.0	0.4 ± 0.0
*Total*	3.2	3.3 ± 0.2	3.4 ± 0.1	3.6 ± 0.1	3.7 ± 0.1	3.6 ± 0.1	3.7 ± 0.1

Mean value ± standard deviation (*n* = 2); significant differences (*p* < 0.05) of a particular parameter in the same ageing year are indicated by different letters.
